# Supplementary material for: Saccharin Supplementation Inhibits Bacterial Growth and Reduces Experimental Colitis in Mice
Source: Nutrients. 2020 Apr 17;12(4):1122. doi: 10.3390/nu12041122 (PMC7230785; doi:10.3390/nu12041122)
Supplement: Supplementary file 1 [file nutrients-12-01122-s001.pdf]

Supplement

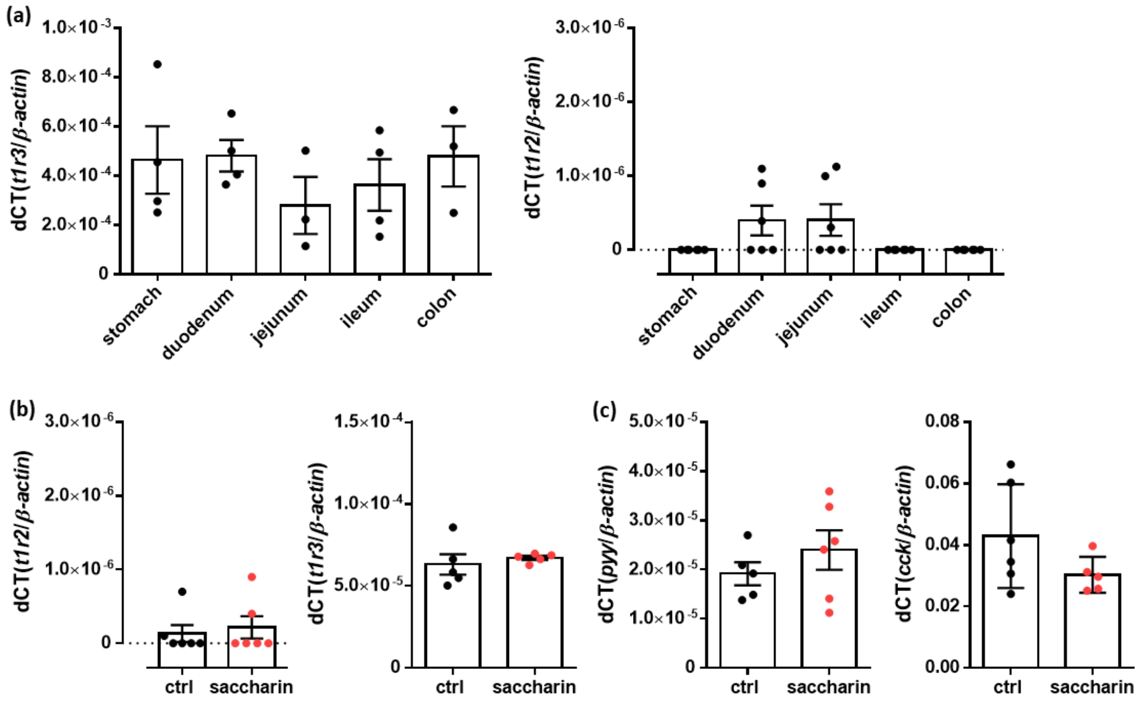

Supplementary Figure 1: *T1r2* and *t1r3* expression in the murine intestine**.** Expression of *t1r3* and *t1r2* was tested in (**a**) different sections of the intestinal tract and in (**b**) proximal jejunum section of seven days saccharin-treated and untreated mice; n=4 and n=6 respectively. Samples with values of zero did not show any expression beyond the detection limit. (**c**) *ß-actin* normalized *pyy* and *cck* mRNA expression levels in biopsies from the proximal jejunum from saccharin-supplemented and ctrl mice; n=6. Values are shown as mean ± SEM.

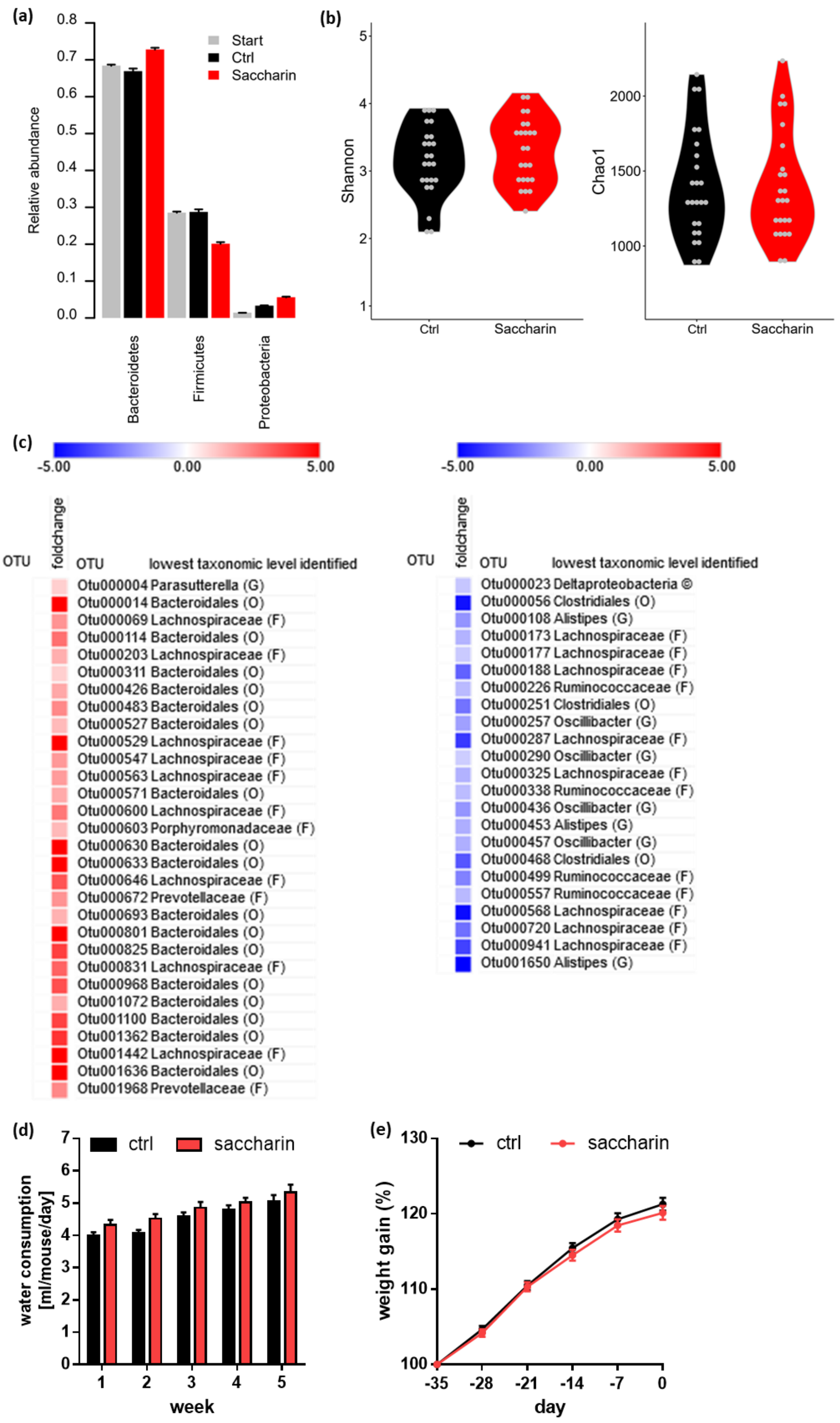
(a)
Relative abundance
0.8
0.7
0.6
0.5
0.4
0.3
0.2
0.1
0.0
Start
Ctrl
Saccharin
Bacteroidetes
Firmicutes
Proteobacteria
(b)
Shannon
Ctrl
Saccharin
Chao1
2000
1500
1000
Ctrl
Saccharin
(c)
-5.00
0.00
5.00
OTU
foldchange
OTU
lowest taxonomic level identified
Otu000004 Parasutterella (G)
Otu000014 Bacteroidales (O)
Otu000069 Lachnospiraceae (F)
Otu000114 Bacteroidales (O)
Otu000203 Lachnospiraceae (F)
Otu000311 Bacteroidales (O)
Otu000426 Bacteroidales (O)
Otu000483 Bacteroidales (O)
Otu000527 Bacteroidales (O)
Otu000529 Lachnospiraceae (F)
Otu000547 Lachnospiraceae (F)
Otu000563 Lachnospiraceae (F)
Otu000571 Bacteroidales (O)
Otu000600 Lachnospiraceae (F)
Otu000603 Porphyromonadaceae (F)
Otu000630 Bacteroidales (O)
Otu000633 Bacteroidales (O)
Otu000646 Lachnospiraceae (F)
Otu000672 Prevotellaceae (F)
Otu000693 Bacteroidales (O)
Otu000801 Bacteroidales (O)
Otu000825 Bacteroidales (O)
Otu000831 Lachnospiraceae (F)
Otu000968 Bacteroidales (O)
Otu001072 Bacteroidales (O)
Otu001100 Bacteroidales (O)
Otu001362 Bacteroidales (O)
Otu001442 Lachnospiraceae (F)
Otu001636 Bacteroidales (O)
Otu001968 Prevotellaceae (F)
-5.00
0.00
5.00
OTU
foldchange
OTU
lowest taxonomic level identified
Otu000023 Deltaproteobacteria ©
Otu000056 Clostridiales (O)
Otu000108 Alistipes (G)
Otu000173 Lachnospiraceae (F)
Otu000177 Lachnospiraceae (F)
Otu000188 Lachnospiraceae (F)
Otu000226 Ruminococcaceae (F)
Otu000251 Clostridiales (O)
Otu000257 Oscillibacter (G)
Otu000287 Lachnospiraceae (F)
Otu000290 Oscillibacter (G)
Otu000325 Lachnospiraceae (F)
Otu000338 Ruminococcaceae (F)
Otu000436 Oscillibacter (G)
Otu000453 Alistipes (G)
Otu000457 Oscillibacter (G)
Otu000468 Clostridiales (O)
Otu000499 Ruminococcaceae (F)
Otu000557 Ruminococcaceae (F)
Otu000568 Lachnospiraceae (F)
Otu000720 Lachnospiraceae (F)
Otu000941 Lachnospiraceae (F)
Otu001650 Alistipes (G)
(d)
ctrl
saccharin
water consumption
[ml/mouse/day]
week
(e)
ctrl
saccharin
weight gain (%)
130
120
110
100
-35
-28
-21
-14
-7
0
day

Supplementary Figure 2: Microbiome analysis, water consumption and weight development of saccharin supplemented or ctrl mice. (**a**) Relative abundance of phyla, (**b**) alpha diversity and (**c**) indicator species in fecal samples obtained after five weeks with or without saccharin supplementation. Heatmap was generated from indicator species analysis with the Morpheus software [48]. In red, taxa being upregulated, in blue, taxa being downregulated in saccharin-treated compared to ctrl mice. Indicator species are listed from most abundant to least abundant OTU. O, order; F, family; G, genus. Start n=48, ctrl and saccharin n=24. (**d**) Water consumption and (**e**) weight development of mice which were supplemented for five weeks with 0.1 mg/ml saccharin via the drinking water or left untreated; n=12 and n=36 from two independent experiments, respectively. (**a**), (**d**), and (**e**) Values are shown as mean ± SEM. (**b**) Values are shown as violin plot.
